#  Fatal Pneumocephalus Caused by Hypervirulent *Klebsiella pneumoniae*, Germany

**DOI:** 10.3201/eid3110.250877

**Published:** 2025-10

**Authors:** Nico Gläser, Nina Schwab, Yvonne Pfeifer, Anika Wahl, Martin A. Fischer, Anja Osterloh, Thomas F.E. Barth, Armin Imhof, Jochen Breitruck, Andreas Essig, Jürgen Benjamin Hagemann

**Affiliations:** Ulm University Hospital, Ulm, Germany (N. Gläser, N. Schwab, A. Osterloh, T.F.E. Barth, A. Imhof, J. Breitruck, A. Essig, J.B. Hagemann); Robert Koch Institute, Wernigerode, Germany (Y. Pfeifer, A. Wahl, M.A. Fischer).

**Keywords:** Pneumocephalus, *Klebsiella pneumoniae*, bacterial infection, bacteria, hypervirulent, meningitis/encephalitis, emerging disease, Germany

## Abstract

We report a fatal case of pneumocephalus in Germany caused by hypervirulent *Klebsiella* *pneumoniae* sequence type 23, confirmed by using clinical, histopathologic, and genomic analyses. The patient reported no travel history, suggesting local emergence. This unusual case reveals an unclear pathogen prevalence and demonstrates the need for increased awareness of global spread.

The global spread of hypervirulent *Klebsiella* *pneumoniae* from Asia poses a major risk to human health that requires constant surveillance (*1*). Those strains are characterized by severe clinical manifestations in previously healthy patients or the presence of convergence plasmids conferring virulence and multidrug resistance that causes difficult-to-treat infections. In this article, we describe a fatal hypervirulent *K. pneumoniae* infection in Germany.

## The Case

A 71-year-old patient was hospitalized with signs of septic shock. Thoracic and abdominal computed tomography (CT) imaging revealed pneumonia and liver and prostate abscesses. We initiated intravenous treatment with piperacillin/tazobactam and clarithromycin. Cranial CT revealed extensive pneumocephalus. We conducted CT by using the SOMATOM Force multidetector (Siemens, https://www.siemens.com) with secondary multiplanar reconstructions and maximum intensity projections. We conducted brain angiography natively and in the arterial phase after contrast medium application, and we conducted thoracic and abdominal imaging in the venous phase.

Three days after hospitalization, the patient died of septic shock with central hypoxia. We isolated hypermucoviscous *K. pneumoniae* from the patient’s blood cultures and postmortem brain samples. The fulminant clinical course justified extensive molecular biologic comparative analyses because similar cases were described previously in Asia (*2*).

We conducted an autopsy after the patient’s death. For postmortem histopathological workup at the Institute of Pathology at Ulm University Hospital (Ulm, Germany), we fixed the patient’s brain in 4% buffered formaldehyde and dissected according to our laboratory protocol. We paraffin-embedded tissue samples from 9 different regions according to our standard laboratory procedures. We conducted conventional (hematoxylin and eosin, Elastica van Gieson, Gram, periodic acid–Schiff, Berliner-Blau [Prussian blue]) and immunohistochemical (CD45, CD68, and GFAP proteins) staining on 3 -µm sections before microscopic evaluation.

We isolated *K. pneumoniae* from blood cultures by using the BACTEC system (BD, https://www.bd.com), postmortem swabs on casein-soy-peptone agar supplemented with 5% sheep blood (Oxoid, http://www.oxoidshop.com), and culture in brain–heart infusion broth (BD) for molecular workup. We used the Bruker (Bruker, https://www.bruker.com) matrix-assisted laser desorption/ionization time-of-flight mass spectrometry system for microbial identification and conducted antimicrobial testing by using VITEK2 (bioMérieux, https://www.biomerieux.com). We then conducted molecular analysis of 2 string test–positive isolates as previously described (*3*). For whole-genome sequencing, we extracted and quantified DNA by using the DNeasy Blood and Tissue Kit (QIAGEN, https://www.qiagen.com) and the Qubit dsDNA HS Assay Kit (Thermo Fisher Scientific, https://www.thermofisher.com). We prepared sequencing libraries by using a Nextera XT DNA Library Prep kit (Illumina, https://www.illumina.com) and conducted sequencing on an Illumina NextSeq 550 (Illumina) by using a v2.5 chemistry kit (2 × 150 bp). We performed genome trimming and assembly as previously described (*3*). We compared the 2 isolates from this patient (0354/24, 0355/25), an additional hypermucoviscous isolate from a patient with liver abscess in the same hospital (0074/24), and 26 recently published *K. pneumoniae* sequence type (ST) 23 isolates from hospitals in Germany (*3*,*4*) by using core genome multilocus sequence typing in SeqSphere 10.0.4 (Ridom, https://www.ridom.de) (*5*). We visualized the resulting tree with iTOL 6.8 (*6*). We identified contigs belonging to possible plasmids by MOBsuite 3.1.4 (*5*,*7*) and predicted resistance and virulence genes and capsular type by using Kleborate v3 (*8*,*9*).

**Figure 1:**
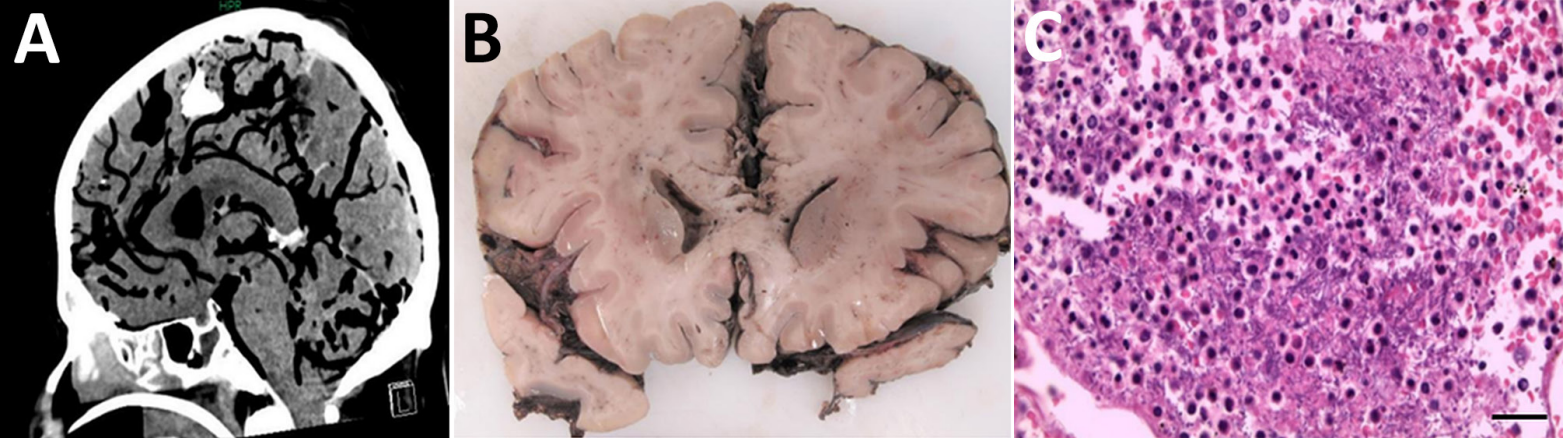
Radiologic and pathologic findings from a fatal pneumocephalus case caused by hypervirulent *Klebsiella pneumoniae* in Germany. A) Computed tomography imaging of the neurocranium revealing gas inclusions both in the arterial and venous system and the inner and outer cerebrospinal fluid spaces consistent with an intravital pneumocephalus. B) Postmortem coronal brain section revealing infiltrates of the leptomeninges and signs of global hypoxic-ischemic brain damage including blurred demarcation between the white and gray matter and discoloration. C) Microscopic appearance of purulent meningitis with bacilli accumulating in the leptomeninges. Hematoxylin and eosin stain. Scale bar indicates 50 µm.

Clinical and radiologic findings supported the diagnosis of sepsis with central nervous system involvement. Although thoracic CT showed bronchial secretions and pulmonary consolidations, cranial CT revealed generalized hypoxic brain damage with intracranial gas inclusions (Figure 1, panel A). Abdominal CT revealed liver and prostate abscesses. On postmortem macroscopic examination, the brain showed signs of global hypoxic-ischemic brain damage, leptomeningitis, diffuse softening, oedematous swelling with signs of herniation (Figure 1, panel B), and numerous parenchymal gas inclusions. Histopathologic findings confirmed global hypoxic-ischemic damage and purulent leptomeningitis (Figure 1, panel C). Gram-stained sections from different brain areas contained gram-negative bacilli consistent with the microbiologic detection of hypermucoviscous *K. pneumoniae* from postmortem brain swab specimens. Hypoxic brain damage because of pneumocephalus caused by hematogenous dissemination of gas-producing bacteria was confirmed as the cause of death. No anatomic or functional anomalies were identified to explain the pneumocephalus. In addition, the autopsy confirmed a chronic liver abscess with incipient connective tissue encapsulation and partly abscessing, phlegmonous prostatitis with proof of *K. pneumoniae*.

**Figure 2:**
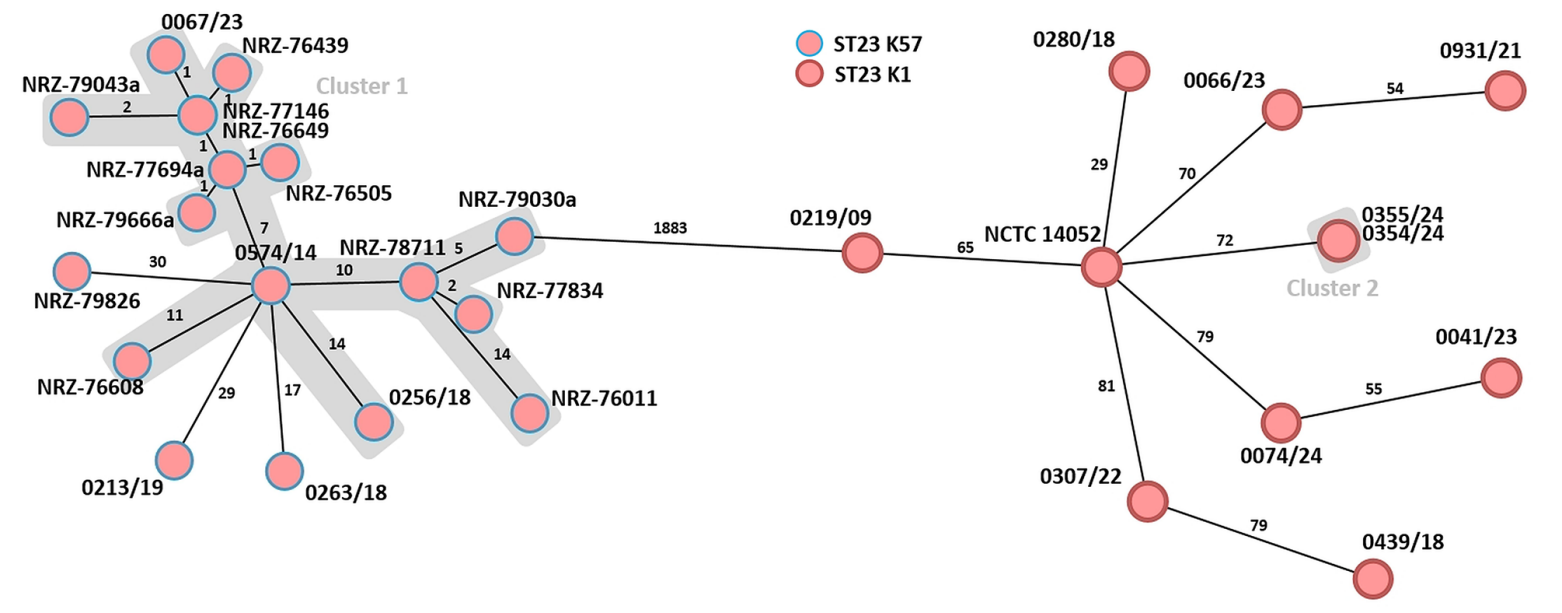
Minimum spanning tree generated to evaluate the genetic relationship of 29 *Klebsiella pneumoniae*-ST23 isolates (capsule types K1 and K57) during study of a fatal pneumocephalus case caused by hypervirulent *K. pneumoniae* in Germany (Appendix Table). Ridom SeqSphere version 10.0.4 (ridom, https://www.ridom.de) was used to create tree for 29 samples isolated from hospitals in Germany on the basis of 2,358 columns, pairwise ignoring missing values, and logarithmic scale. Distance is on the basis of columns from *K. pneumoniae* sensu lato core genome multilocus sequence type (2,358 genes). Cluster distance threshold is 15 according to the Ridom Seqsphere. The patient isolates from this case (0354/24 and 0355/24) are labeled as cluster 2. Isolate 0074/24 is another *K. pneumoniae* ST23-K1 isolate from a patient with liver abscess admitted to the same hospital. Cluster 1 represents *K. pneumoniae* ST23-K57 isolates with virulence or convergence plasmids that were in part from patients previously hospitalized in Ukraine (*3*,*4*). ST, sequence type.

**Figure 3:**
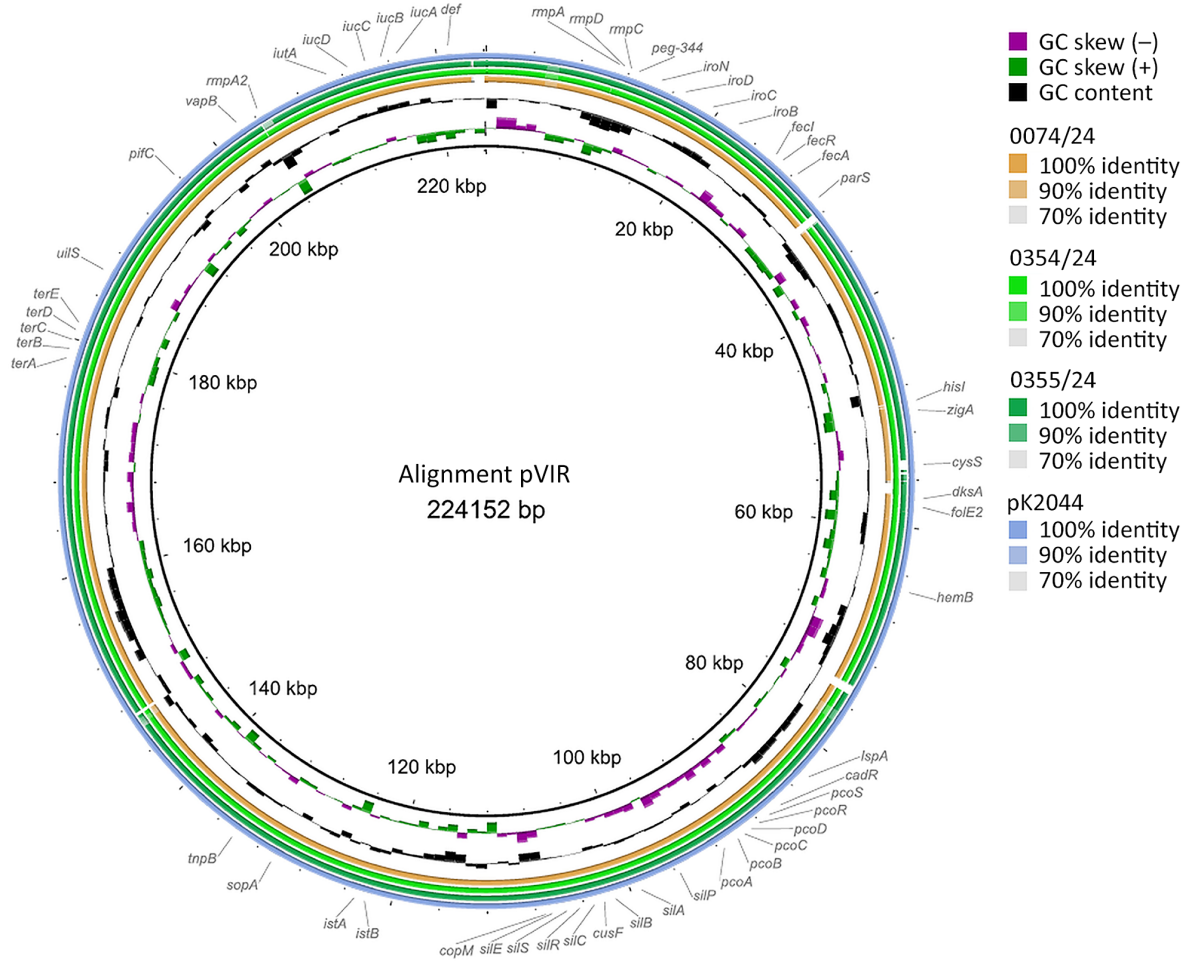
Comparative plasmid alignment of hypervirulent *Klebsiella pneumoniae* from the study of a fatal pneumocephalus case in Germany, completed by using BRIG (*10*). The innermost rings show guanine-cytosine content, and the outer ring shows the used reference virulence plasmid pK2044 (blue) from *K. pneumoniae* strain NTUH-K2044 from Taiwan (*11*). Concentric rings illustrate the virulence plasmids of the *K. pneumoniae* isolates from this case, 0354/24 (light green) and 0355/24 (dark green), and a patient isolate from the same hospital, 0074/24 (orange). Sequence similarity to the reference plasmid is indicated by the intensity and continuity of the colored rings. The gaps correspond to regions with no major sequence similarity. The locations of the typical virulence-associated genes (*iucA, iroB, rmpA, rmpA2, peg344*) (*12*) are visualized on the outermost ring.

Genome analyses of the 2 hypermucoviscous *K. pneumoniae* isolates from blood cultures detected the *magA* gene, which is specific for capsule type K1, and a characteristic combination of virulence-associated genes (*iucA*, *iroB*, *peg-344*, *rmpA*, and *rmpA2*) (*10*). Multilocus sequence typing assigned both isolates to ST23. Subsequent comparison with 27 other *K. pneumoniae* ST23 isolates from Germany with capsule types K1 and K57 revealed that the isolates from this case were genetically identical (minimum spanning tree cluster 2) but not closely related to the other isolates (Figure 2). Plasmid analysis identified a type AA406 virulence plasmid in the 2 isolates from this study and 25 of the 27 other ST23 isolates (Appendix Table). An alignment of the plasmids with the characteristic Pl virulence plasmid pK2044, which is almost exclusively found in ST23 isolates, confirmed a high sequence similarity (Figure 3) (*10**,**11*). In addition, 4 previous ST23-K57 isolates carried a convergence plasmid (AA405) containing both virulence-associated and antimicrobial resistance genes, highlighting the emerging clinical significance of ST23 (*3*,*4*).

## Conclusions

We describe a lethal pneumocephalus case caused by hypervirulent *K. pneumoniae* ST23 in Germany. Similar cases were reported from Southeast Asia (*13*). To date, we have found only sporadic reports of hypervirulent and mostly imported *K. pneumoniae* strains in Europe (*3*). Of note, our patient had no history of travel, and his only well-described risk factor was type 2 diabetes mellitus. Poor glycemic control increases the risk for fulminant *K. pneumoniae* infections with hematogenous dissemination; mortality rates of meningitis in such cases exceed 50% (*1*,*14*). 

Despite early intensive care and antiinfective treatment, this patient died within 72 hours of hospitalization. The pathogenesis of the fulminant pneumocephalus is likely the consequence of septic dissemination from his primary infection site to the central nervous system with subsequent gas production. The patient’s *K. pneumoniae* ST23 isolates contained a virulence plasmid of type AA406 with typical virulence-associated genes and demonstrated no close genetic link to earlier reported *K.* *pneumoniae* ST23 strains in Germany (Figures 2,3). 

This case indicates a potentially underestimated prevalence of hypervirulent ST23 strains in Europe, which requires systematic surveillance to better assess the distribution and epidemiology of *K. pneumoniae* ST23 (*15*) (https://op.europa.eu/en/publication-detail/-/publication/1e194058-d79e-11ee-b9d9-01aa75ed71a1). Clinicians should consider hypervirulent strains in cases of severe or recurrent *K. pneumoniae* infections, even without prior geographic exposure, and rapidly initiate microbiological diagnostics. Because not all hypervirulent *K. pneumoniae* strains are hypermucoviscous, and not all hypermucoviscous strains are hypervirulent (*3*), further molecular analyses should be implemented when hypervirulent *K. pneumoniae* infection is suspected. If molecular analysis is not possible despite clinical suspicion of infection with a hypervirulent strain, clinical management should be adjusted quickly (e.g., identification of occult abscesses). 

Because of the dynamic global spread of pathogens, clinical and laboratory diagnostics are critical to efficient surveillance strategies for emerging pathogens such as hypervirulent *K. pneumoniae*, the epidemiologic relevance of which might still be underestimated. Further research is needed to clarify the reasons for different clinical courses in infections with hypervirulent *K. pneumoniae* isolates despite harboring identical virulence factors.

Against the background of globalization, complex trading networks, and increasing travel activities, enhanced genomic surveillance is crucial to detect and respond to novel pathogen threats such as hypervirulent *K. pneumoniae*. Close interdisciplinary collaboration between clinicians, pathologists, epidemiologists, and microbiologists is essential for a comprehensive understanding and sound surveillance practices.

Appendix Additional information about fatal pneumocephalus caused by hypervirulent *Klebsiella pneumoniae*, Germany.
